# Rectal carcinoma with dual differentiation toward enteroblastic and neuroendocrine features arising in a patient with ulcerative colitis: a case report

**DOI:** 10.1186/s12957-022-02838-1

**Published:** 2022-11-26

**Authors:** Takako Kihara, Ryuichi Kuwahara, Kurando Kusunoki, Tomohiro Minagawa, Yuki Horio, Motoi Uchino, Hiroki Ikeuchi, Seiichi Hirota

**Affiliations:** 1 Department of Surgical Pathology, Hyogo Medical University Hospital, 1-1 Mukogawa-cho, Nishinomiya, Hyogo 663-8501 Japan; 2 Division of Inflammatory Bowel Disease Surgery, Department of Gastroenterological Surgery, Hyogo Medical University Hospital, 1-1 Mukogawa-cho, Nishinomiya, Hyogo 663-8501 Japan

**Keywords:** Colorectal carcinoma, Carcinoma with enteroblastic differentiation, Carcinoma with neuroendocrine differentiation, Ulcerative colitis

## Abstract

**Background:**

Colorectal carcinoma with enteroblastic differentiation is a rare subtype of colorectal carcinomas expressing at least one characteristic immunohistochemical marker among *α*-fetoprotein, glypican-3, and spalt-like transcription factor 4. On the other hand, colorectal carcinoma with neuroendocrine differentiation is also a unique subtype of colorectal carcinomas showing expression of at least one distinctive marker among chromogranin A, synaptophysin, and CD56.

**Case presentation:**

We experienced an extremely rare case of rectal carcinoma with dual differentiation toward enteroblastic and neuroendocrine features in a 53-year-old male patient with long-standing ulcerative colitis (UC). Most of the tumor cells were positive for enteroblastic differentiation markers and approximately a half of them for neuroendocrine differentiation markers. Some tumor cells showed only enteroblastic differentiation, and some did only neuroendocrine feature, but some showed both enteroblastic and neuroendocrine differentiation.

**Conclusion:**

Colorectal carcinoma with dual differentiation toward enteroblastic and neuroendocrine features has not been reported yet. Neoplastic transformation from pluripotent stem cells in dysplastic epithelium of long-standing UC patients may be associated with such dual differentiation features.

## Background

Carcinomas with enteroblastic differentiation (ED) are a rare variant of carcinomas with histological features of clear cytoplasm like embryonal gastrointestinal (GI) epithelia and tubulopapillary or solid growth pattern. They characteristically express enteroblastic markers such as α-fetoprotein (AFP), glypican-3 (GPC3), and spalt-like transcription factor 4 (SALL4). To our knowledge, approximately 80 cases of primary colorectal carcinoma (CRC) with ED including colorectal clear cell carcinoma have been reported to date [1–8].

CRCs with neuroendocrine differentiation (NED) or colorectal neuroendocrine carcinomas are also a rare type of CRCs accounting less than 1% of all CRCs [9]. According to the 2022 WHO Classification of Neuroendocrine Neoplasms, neuroendocrine neoplasms (NENs) with both epithelial and neuroendocrine tumor components in all organ systems are called mixed neuroendocrine-non-neuroendocrine neoplasms (MiNENs) [9]. NENs associated with UC might develop, and 38 cases of CRC with NED in UC patients have been reported to date [10–16].

To our knowledge, however, CRC with both enteroblastic differentiation and neuroendocrine differentiation has not been reported yet in the English literature. We describe here the first case of such rectal carcinoma in a 53-year-old male patient with long-standing UC.

## Case presentation

A 53-year-old Japanese man suffered from UC for 34 years. He was initially diagnosed with UC in 1987 when he was 19 years old. Medications including oral prednisolone and azathioprine were transiently effective, but the disease frequently relapsed. During the follow-up, a surveillance colonoscopy with biopsy was repeated, and the recent colonoscopy revealed a flat lesion in the lower rectum, and rectal biopsy was done. Specimen of the rectal biopsy was diagnosed as adenocarcinoma. He admitted to our hospital for treatment for the long-standing UC with rectal cancer. A total colectomy with the ileoanal anastomosis was performed. He has no recurrency for 4 months after the surgery.

**Fig. 1:**
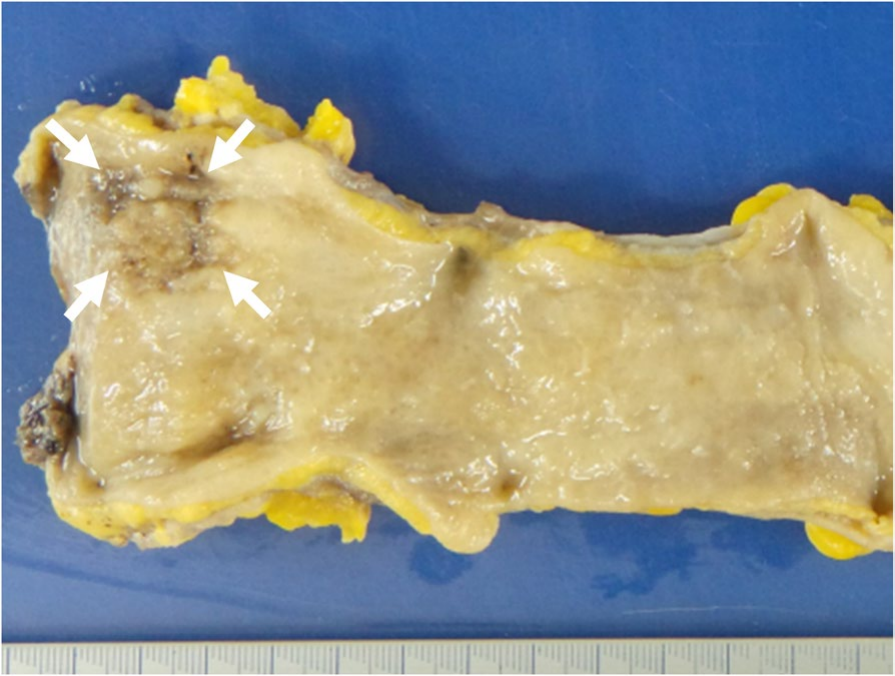
Macroscopic findings of the resected flat 2.5 × 2.5 cm tumor in the lower rectum. Arrows indicate the lesion

**Fig. 2:**
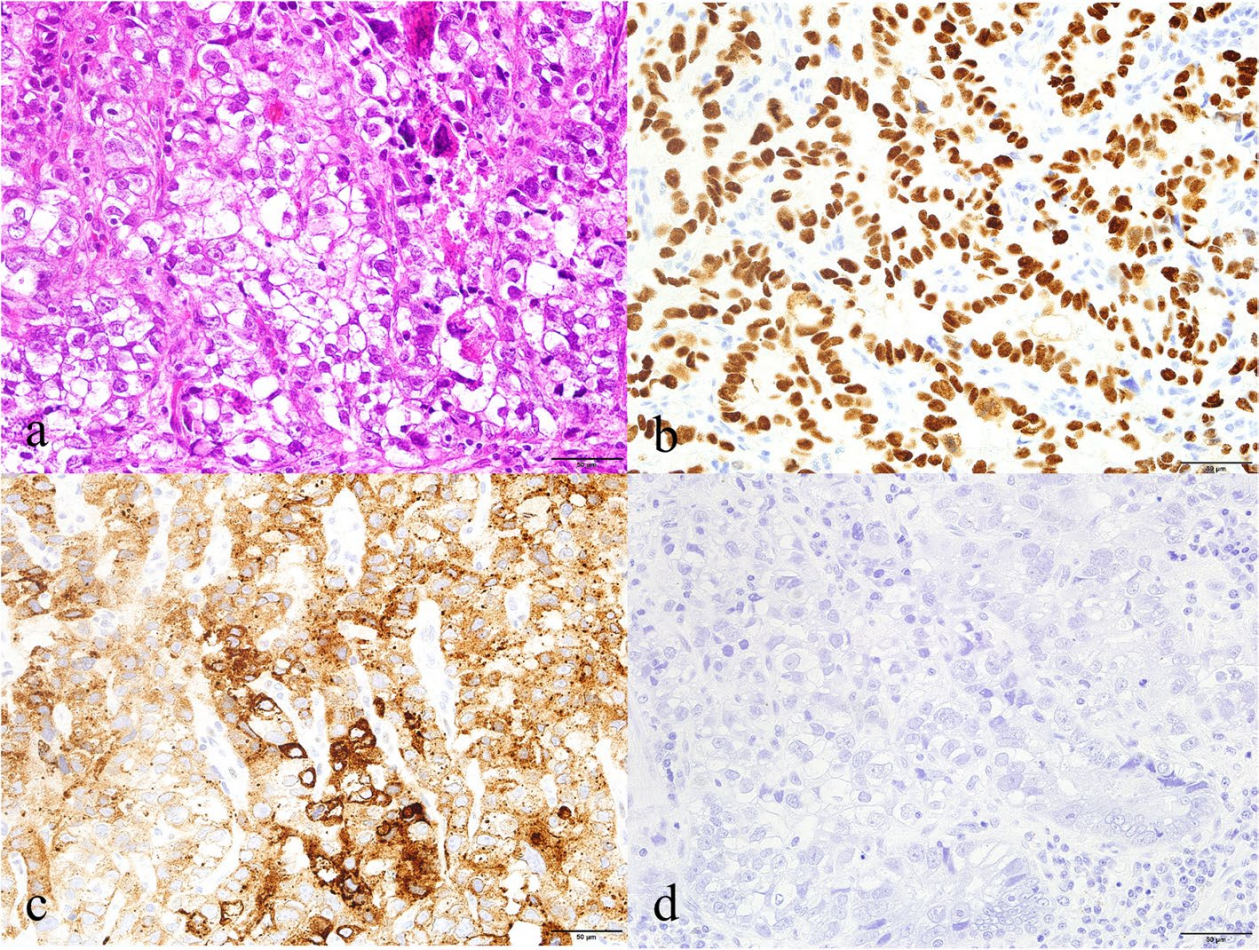
Representative histology of the rectal carcinoma with ED. **a** The tumor was composed of predominantly moderately differentiated adenocarcinoma with focal clear cell features (H&E × 400). Immunohistochemistry revealed that the tumor cells with clear cytoplasm and prominent nucleoli were positive for nuclear SALL4 (**b**) and cytoplasmic GPC3 (**c**) but negative for AFP (**d**) (× 400)

**Fig. 3:**
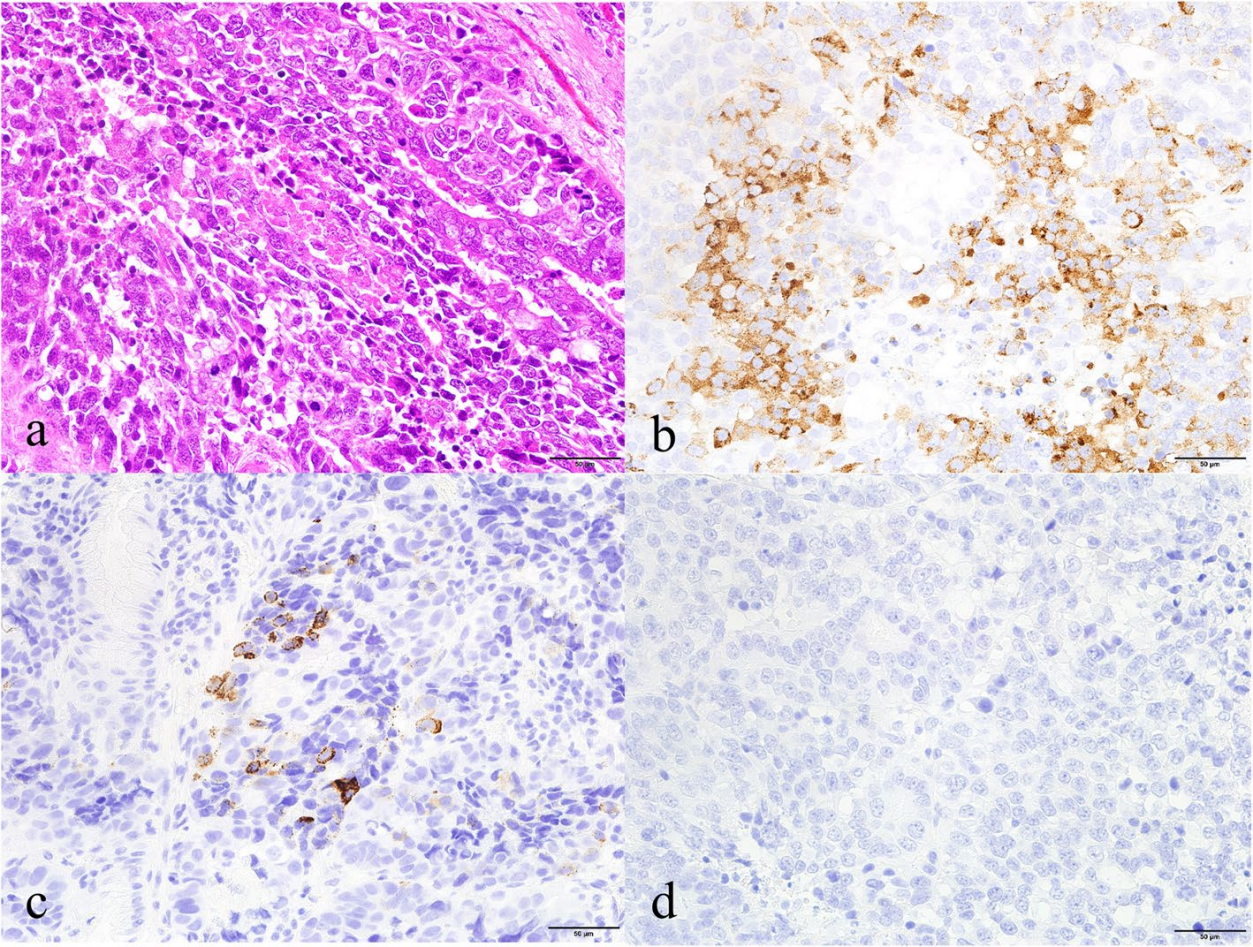
Representative histology of the rectal carcinoma with NED. The tumor was composed of predominantly poorly differentiated carcinoma with focal necrosis (**a**, H&E × 400). Immunohistochemistry revealed that the tumor cells forming solid nests were positive for cytoplasmic synaptophysin (**b**), focally positive for cytoplasmic chromogranin A (**c**), but negative for CD56 (**d**) (× 400)

**Fig. 4:**
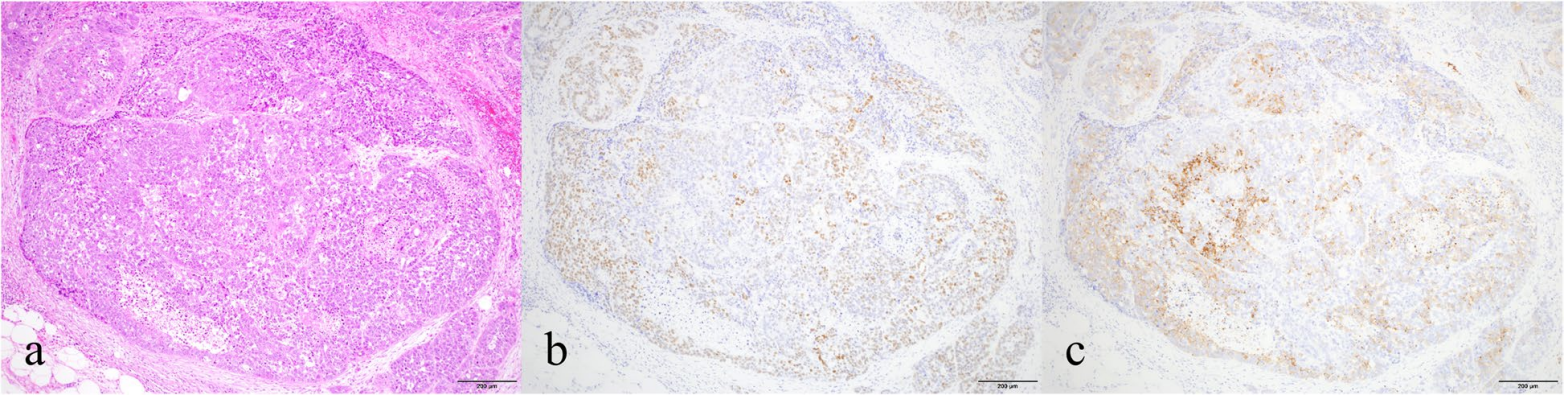
Representative histology of rectal carcinoma showing dual differentiation toward enteroblastic and neuroendocrine features in the same cells. **a** Tumor cells partially arranged in glandular patterns and partially showed solid growth (H&E × 40). SALL4 (**b**) was immunohistochemically positive in a part of the tumor cells, and synaptophysin (**c**) was also positive in a part of them. Expression of SALL4 (**b**) and synaptophysin (**c**) was overlapping in some tumor cells

Grossly, the resected flat tumor of the lower rectum was 2.5 × 2.5 cm in size (Fig. 1). Histological examination showed that the tumor had a component of moderately differentiated tubular adenocarcinoma with focal clear cytoplasm (Fig. 2a). The tumor cells predominantly arranged in glandular pattern, and most had hyperchromatic nuclei and prominent nucleoli (Fig. 2a). Immunohistochemistry revealed that the clear cells were positive for both GPC3 (Fig. 2b) and nuclear SALL4 (Fig. 2c), indicating enteroblastic differentiation. They were negative for AFP (Fig. 2d). In addition to the glandular growth pattern, tumor had a component of solid and nested growth pattern (Fig. 3a). Those tumor cells were positive for synaptophysin (Fig. 3b) and focally positive for chromogranin A (Fig. 3c), indicating neuroendocrine differentiation. They were negative for CD56 (Fig. 3d). Thus, some tumor cells showed only enteroblastic differentiation, and some tumor cells did only neuroendocrine feature, but other tumor cells were positive for both enteroblastic differentiation markers and neuroendocrine differentiation markers, indicating amphicrine cells (Fig. 4a, H&E; b, SALL4 immunohistochemistry; c, synaptophysin immunohistochemistry). Some tumor cells are both negative for enteroblastic differentiation markers and neuroendocrine differentiation markers (Fig. 4a, H&E; b, SALL4 immunohistochemistry; c, synaptophysin immunohistochemistry). Diffuse and strong staining for p53 was observed in most of the tumor cells (data not shown), suggesting the presence of TP53 mutation. Ki-67 labeling index of the tumor cells was more than 90% (data not shown). The tumor cells infiltrated into the submucosal layer with lymphatic invasion and venous invasion. One out of the five dissected lymph nodes showed metastasis. No distant metastasis was found by imaging tests. Thus, the tumor staging was regarded as pT1bN1a(1/5)M0 according to TNM classification [17]. The mucosa (5 × 3 cm) adjacent to the flat tumor showed low-grade dysplasia.

## Discussion

Patients with long-standing UC have a high risk of CRC. The risk of developing CRC increases up to 2% after 10 years, 8% after 20 years, and 18% after 30 years [18]. The most frequent type of CRC associated with UC is adenocarcinoma, but other types of carcinoma such as squamous cell carcinoma, small cell carcinoma, and “hepatoid” carcinoma have been described [19]. In the present report, we described the first case of CRC with dual differentiation toward enteroblastic and neuroendocrine features in a patient with long-standing UC.

In our case, components of both enteroblastic differentiation and neuroendocrine differentiation were rather widely observed in the rectal carcinoma. Guadagno et al. described a case of incidental neuroendocrine microcarcinoma coexistent with a high-grade adenoma in the rectum, in which distant metastases of the neuroendocrine carcinoma component occurred in a few months [20]. Since neuroendocrine carcinoma component should be considered extremely malignant even if the lesion is very small, we should accurately differentiate the neuroendocrine carcinoma component from usual solid-type adenocarcinoma through the appropriate immunohistochemical examination. The careful follow-up is also needed in our case.

UC-associated carcinoma is considered to develop from dysplasia through a pathway called inflammation-dysplasia-carcinoma sequence [21]. Long-standing inflammation may cause “pancellular damage” involving all types of colonic epithelial cells, probably resulting in development of tumor with NED derived from pluripotent stem cells in dysplastic epithelium. Shigaki K. et al. observed that about 30–50% of UC-associated dysplasia had a feature of NED, suggesting that the multipotential cell might be capable of giving rise to neoplasia with NED [22]. Thus, pluripotent stem cells in dysplastic epithelium of long-standing UC patients may differentiate not only to neuroendocrine features but also to enteroblastic features. In 2020, Yamashiro Y. et al. identified 39 (4.01%) out of 971 CRC cases which was immunohistochemically positive for at least one enteroblastic marker, but only approximately one fourth of them contained tumor cells with clear cytoplasm [23]. Apparent clear cytoplasm of the tumor cells as in the present case could be reminiscent of ED, but the enteroblastic features in CRC may be rather overlooked when the tumor cells do not have obviously clear cytoplasm and is observed only by H&E staining. Immunohistochemical staining of AFP, GPC3, and SALL4 in many cases of CRC in UC patients may clarify whether quite a few CRC in UC patients might show ED.

## Conclusion

We reported the first case of CRC with dual differentiation toward enteroblastic and neuroendocrine features. Background of long-standing UC may be associated with such features.

## Data Availability

All data supporting the funding of this study are available within the article.

## Abbreviations

UC: Ulcerative colitis
ED: Enteroblastic differentiation
GI: Gastrointestinal
AFP: *α*-Fetoprotein
GPC3: Glypican-3
SALL4: Spalt-like transcription factor 4
CRC: Colorectal carcinoma
NED: Neuroendocrine differentiation
NENs: Neuroendocrine neoplasms
MiNENs: Mixed neuroendocrine-non-neuroendocrine neoplasms
